# Location Privacy for Mobile Crowd Sensing through Population Mapping ^†^

**DOI:** 10.3390/s150715285

**Published:** 2015-06-29

**Authors:** Minho Shin, Cory Cornelius, Apu Kapadia, Nikos Triandopoulos, David Kotz

**Affiliations:** 1 Myongji University, Myongjiro 116, Yongin 449-728, Korea; 2 Intel Labs, Hillsboro, OR 97124, USA; E-Mail: cory.cornelius@intel.com; 3 Indiana University, Bloomington, IN 47408, USA; E-Mail: kapadia@indiana.edu; 4 Boston University, 111 Cummington Mall, Boston, MA 02215, USA; E-Mail: nikos@cs.bu.edu; 5 Dartmouth College, Hanover, NH 03755, USA; E-Mail: kotz@cs.dartmouth.edu

**Keywords:** location privacy, *k*-anonymity, mobility traces

## Abstract

Opportunistic sensing allows applications to “task” mobile devices to measure context in a target region. For example, one could leverage sensor-equipped vehicles to measure traffic or pollution levels on a particular street or users' mobile phones to locate (Bluetooth-enabled) objects in their vicinity. In most proposed applications, context reports include the time and location of the event, putting the privacy of users at increased risk: even if identifying information has been removed from a report, the accompanying time and location can reveal sufficient information to de-anonymize the user whose device sent the report. We propose and evaluate a novel spatiotemporal blurring mechanism based on tessellation and clustering to protect users' privacy against the system while reporting context. Our technique employs a notion of probabilistic *k*-anonymity; it allows users to perform local blurring of reports efficiently without an online anonymization server before the data are sent to the system. The proposed scheme can control the degree of certainty in location privacy and the quality of reports through a system parameter. We outline the architecture and security properties of our approach and evaluate our tessellation and clustering algorithm against real mobility traces.

## 1. Introduction

The concept of a smart city can provide high-quality services to the citizens and reduce its operational costs by information and communication technology [1]. By sensing and analyzing the urban environment, a smart city can make intelligent decisions on satisfying various needs in the city, including daily livelihood, environmental protection, public safety and city services and industrial and commercial activities [2]. By leveraging citizen's mobile devices to measure environmental context, mobile crowd sensing becomes a central part of any smart city.

Mobile crowd sensing has been gaining popularity, with several systems and applications being proposed to leverage users' mobile devices to measure environmental context. Applications of mobile crowd sensing include monitoring city noise [3,4], city climate [5], people density [6], emergency behavior [7], traffic anomalies [8] and even detecting earthquakes [9]. These systems predominantly rely on mobile nodes whose carriers are humans (or their personal vehicles) in an urban environment, thus putting the privacy of users at risk. For example, location and time, which are often included as context in sensor reports, reveal the movements of the reporting users.

In many mobile crowd-sensing applications, knowing the identities of the devices is unnecessary. Indeed, to protect users' privacy, Tang *et al.* [10] take the approach of suppressing the node's identity from reports, and Calandriello *et al.* [11], PEPSI [12], and AnonySense [13] support pseudonymous or anonymous reports. Unfortunately, knowing a user's locations (coupled with the knowledge of their historical movement patterns) is often enough to de-anonymize their reports [14]. A report taken inside Alice's office, for example, allows one to infer that Alice was likely in her office, even if her name was suppressed from the report. More complicated attacks against pseudonymized data are described by Chow *et al.* [15].

Borrowing from the data privacy literature, the principle of *k*-anonymity [16] has been used to preserve the location privacy of mobile users [15,17–25]. Location *k*-anonymity provides a form of plausible deniability by ensuring that the user cannot be individually identified from a group of *k* users who have appeared at a similar location and time. This can be achieved by blurring the location and time of the user so that there exist at least *k* −1 other users with the same locations and time. In this paper, we employ this *k*-anonymity principle through spatio-temporal blurring of the reports to protect the location privacy of reporting users.

Prior approaches to location blurring require a dedicated and high-performance centralized server [18,19,21] or complex and time-consuming peer-to-peer communication [15,25,26]. The centralized approach has shortcomings: (i) the anonymization server becomes the performance bottleneck or single point of failure; and (ii) the anonymization server, if compromised, can pose a serious threat to the privacy of all of the users. On the other hand, the peer-to-peer approach requires local multi-party communication between peers and needs a high level of trust amongst peer nodes (for example, the user needs to trust peer nodes to not disclose the locations of the user).

To address the limitations of the existing approaches, we propose a novel location-privacy mechanism that exhibits three distinguishing properties. First, each mobile node (MN) infers its own level of location privacy (self-discernment). Second, the privacy guarantee is only probabilistic (probabilistic *k*-anonymity), adding flexibility and efficiency. Third, the mechanism is efficient to perform.

In our approach, the MN computes the k-anonymous region based on a population map provided by the map server (MS). The population map, however, provides only a probabilistic estimation of anonymity levels, which introduces the novel notion of probabilistic *k*-anonymity, denoted by (*k*, *p*)-anonymity. A user holds (*k*, *p*)-anonymity if the user is expected to enjoy *k*-anonymity with probability at least *p*. In other words, with at most 1 − *p* probability, the user may belong to an anonymity-set smaller than *k*.

Our user-driven probabilistic location privacy scheme provides better performance and few trust assumptions compared to existing work. The scheme achieves better performance by removing the need of an online blurring server. Instead, the map server performs background data collection to build history-based population density maps, and users occasionally obtain up-to-date (*k*, *p*)-anonymous region maps from the server. Unlike the on-line blurring servers found in prior work, the role of the map-server is limited to off-line computations, and thus, it requires fewer trust assumptions regarding user privacy. For example, the map server is not responsible for blurring each report before forwarding it to the report server in real time and, thus, cannot (maliciously) manipulate the level of privacy provided by each report.

We make the following contributions:

- We propose a novel notion of probabilistic privacy, called (*k*, *p*)-anonymity, and demonstrate the concept in mobile crowd sensing.
- We develop a new automatic local blurring technique that blurs the locations of the reports based on a map built under the probabilistic *k*-anonymity principle.
- We evaluate our approach based on real mobility traces representing 730,943 wireless access-point associations over 21 days and show that a reasonable trade-off can be achieved between users' privacy and the spatiotemporal granularity of the reports.

In the following sections, we describe some use cases of mobile crowd sensing in a smart city, present our security model, then describe our tessellation-based technique in Section 4, followed by an evaluation of the privacy provided by tessellation in Section 5. We discuss several issues in Section 6 and review related work in Section 7. We conclude in Section 8.

## 2. Use Cases

As a general motivation for the use of anonymous people-centric sensing systems, we describe four example applications in the context of smart cities; namely environmental sensing, object sensing, traffic monitoring and infrastructure maintenance. In later sections, these use cases provide context for estimating reasonable system parameters and for evaluating our approach.

### 2.1. Environmental Sensing Applications

There is great interest in sensing environmental conditions: at the global scale for climate-change modeling, at the urban scale for studies in public health and urban planning and at the building scale to augment personal health monitoring and to address issues like sick building syndrome [27]. Anonymous people-centric sensing may be particularly useful in monitoring urban micro-climates.

The “UScan” study made fine-grained temperature measurements in downtown Tokyo by deploying 200 stationary sensor nodes in an area of 107,500 m^2^, with each node sensing an area of 537 m^2^, roughly equivalent to a circle of a radius of 13 m [5]. This study is one of the few large-scale urban-scale temperature-monitoring networks and has the highest node density to date. Other studies are focusing on non-urban areas or on urban areas, but with fewer nodes. (for example, one network includes 165 sensor nodes in a national park spanning 583,000 m^2^, with each node covering a circle of a radius of 33.5 m [28].) Such networks are helpful in studying phenomena like urban heat islands, although scientists need high node density and large coverage area, which is expensive and logistically difficult if stationary sensor nodes must be installed and maintained across an urban area.

With the development of appropriate sensors (such as temperature, humidity, air pressure, air quality) and appropriate algorithms for detecting when a smart phone is capable of obtaining a relevant measurement (out of pocket and outside a building), it may be possible to collect urban micro-climate information from mobile phones and volunteer users. With millions of phones circulating in most large cities, it may be possible to get the high density and large coverage scientists need, with a few stationary nodes deployed for correlating mobile sensors with a ground truth.

### 2.2. Object-Sensing Applications

In addition to sensing natural phenomena, it can be of great use if devices can sense everyday objects. For example, shopping can be streamlined if shoppers know where interesting products are located in the store, and a lost key chain could be located quickly before someone picks it up. Most object-sensing systems require expensive infrastructure, such as radio frequency identification (RFID) readers installed in the environment. Recently, however, mobile-phone based object-sensing systems have been proposed.

ObjectFinder [13,29] can request other mobile phones in a certain area to find a Bluetooth-enabled object. When a mobile phone detects the specified MAC address, it then reports the current location. The app is then able to mark on a map where the target object was detected. Although the positioning may be crude, one could easily imagine ObjectFinder being extended to include other information, such as signal strength, so triangulation can be used for more accurate object positioning.

### 2.3. Traffic Monitoring Applications

Current traffic monitoring systems use fixed-position traffic sensors (using either roadside cameras or magnetic loops embedded in the ground) to monitor traffic volume. However, the high cost of the equipment and its installation makes such a system available only on major roads. Now that many people carry mobile phones with a GPS receiver, mobile-phone-based traffic monitoring systems are gaining attention due to their low cost and large coverage (e.g., the Mobile Millennium Project by UC Berkeley [30]).

In a cellphone-based traffic monitoring system, each participating mobile phone reports its travel information to a backend server, which analyzes thousands of reports and provides the users with an estimated travel time. As it travels, each phone may report it GPS readings [31] or its travel time along predefined road segments. Studies have focused on how to build such a system, how to estimate travel times from mobile traffic measurements and how various operation parameters affect the accuracy of the system. Other studies have used the phone's sensors to detect potholes, noise and road conditions [32,33]. However, the privacy of reporting users is one of the biggest challenges for such systems [31,34].

### 2.4. Infrastructure Monitoring

Consider the other city infrastructure a pedestrian might encounter: streetlights, crosswalks, bike racks, park benches, trash receptacles, signage and public buildings. A city keen to maintain its infrastructure may benefit from crowdsourcing reports of broken or inoperative infrastructure. We envision a smartphone application that allows mobile citizens to photograph or otherwise record problematic points and upload them, anonymously, for the city's maintenance unit. In these cases, it may suffice to approximate location on the order of a city block and time on the order of one day.

## 3. Security Model

In what follows, we describe our sensing model, threat model, privacy model, trust assumptions and desired security properties.

### 3.1. Sensing Model

In a system for mobile crowd sensing (for example [35]), the system collects and processes sensed data from a large number of personal devices (e.g., smart phones) carried by the users. The devices are equipped with sensors and have at least intermittent network connectivity (e.g., via Wi-Fi). These tasked mobile nodes (MN) report sensor data to the application via the network; these reports usually include a timestamp and location information. Users, whom we call carriers, participate in the system by enabling their devices to perform sensing tasks on behalf of others who submit the tasks. The tasking person runs an application to generate and submit a task, either from a computer or from a mobile device.

A carrier may be motivated to participate in a sensing task for many reasons. They may be mandated to participate; for example, a company may require their employees (who use company-provided mobile phones) to report atmospheric measurements in their vicinity to improve the air quality in the facility. They may be provided incentives; for example, a university may motivate their students to report movement patterns for the purpose of modeling pedestrian traffic, by rewarding them with cafeteria credit points. Finally, they may simply volunteer for the public good; for example, a municipal organization may want to monitor noise pollution levels in the metro area from pedestrians who are willing to contribute their devices to make occasional readings.

Within the device, sensing activities are performed by a sensing software. This software is distributed by the sensing system, by a third party or even by the carrier. The sensing software may be dedicated for one sensing task (e.g., traffic monitoring [30]) or may be a generic sensing framework that can perform arbitrary sensing tasks [13,35]. In either case, the software submits reports (*t*, *l*, *d*) containing sensed data *d* and a time-location pair (*t*, *l*) to a backend server when the network is available [13,36].

### 3.2. Threat Model

We consider an adversary who wants to learn the victim's location and time pairs from the sensing reports submitted by the mobile nodes. The adversary can be a malicious application, which submits a sensing task and receives corresponding reports, or an intruder to the sensing system, which gains access to all of the reports.

Since the report itself does not have any identifying information, the location and time component of the report is the only clue for the attacker (as other fields are environmental sensor readings). Moreover, the attacker needs external knowledge about the carrier, such as significant places or typical movement patterns. With such external knowledge, the time-location pair in the report serves as a quasi-identifier attribute [16] that helps to identify the victim's reports; for example, “David usually arrives at building X at 8 a.m. and then leaves for building Y at 10 a.m. for the class”. This known pattern can be used to identify some of David's reports. Starting from these reports, the adversary may attempt to track David's further reports to discover his next destination. For location tracking, the adversary needs to identify multiple reports as originating from the same person [15,37].

### 3.3. Privacy Model

We base our privacy model on the concept of *k*-anonymity, *i.e.*, from the adversary's perspective, any report should appear to have come from one out of *k* possible people. In this section, we discuss how we model our privacy-aware mobile crowd sensing problem and how it relates to the *k*-anonymity principle as originally defined by Sweeney [16].

The *k*-anonymity model [16] was developed in the context of micro-data publishing, where tuples regarding individuals are published. Although a tuple may contain no identity attributes, such as a social security number, some attributes, when combined with some external knowledge, may collectively identify the user or narrow down to a handful set of tuples that may belong to the victim. Such attributes are called quasi-identifier attributes (QA). The *k*-anonymity principle generalizes each QA to ensure that every QA-instance appears at least *k* times in the dataset. As a result, the adversary cannot narrow down the anonymity set to fewer than *k* tuples, keeping the victim anonymous among *k* individuals.

We model the privacy-aware mobile crowd sensing problem as a variant of the privacy-aware micro-data publishing problem. In mobile crowd sensing, each carrier wants to publish its sensor reports. If published as-is, the fine-grained location information therein (serving as QA) can reveal the carrier's location trace (see Section 3.2). Therefore, before publishing, each carrier generalizes (*i.e.*, blurs) the time and location attributes within the reports so that there are *k* − 1 other carriers with the same blurred time-location pair.

However, our model differs from the original data-publishing problem. First, although every carrier joins the blurring process, whether they actually publish their reports depends on various factors: whether the carrier is participating in a sensing task, whether the task requires a report at this moment, whether a network connection is available for report submission, and so on. That is, each carrier is only a potential individual in the dataset. Second, each carrier publishes its data multiple times as long as there are reports to submit.

Note that the above two distinguishing characteristics of mobile crowd sensing make the adversary's attack method more complicated. That is, even if there exists only one report observed at a specific time and location, the adversary should admit that there are potentially *k* different carriers that may be the reporting carrier, when the blurring scheme for *k*-anonymity is present in the reporting process. Moreover, the adversary is not sure if two separate reports belong to the same carrier or to two different carriers.

In this paper, we aim to provide *k*-anonymity regarding location privacy in the context of privacy-aware mobile crowd sensing as described. However, we only seek to guarantee the privacy goal probabilistically. We choose a probabilistic approach as a consequence of our architectural choices that we make on behalf of performance and security. This probabilistic *k*-anonymity, which we call (*k*, *p*)-anonymity, provides *k*-anonymity with probability of at least *p*. Conversely, with probability of 1 − *p*, the anonymity set size may be less than *k*. Despite this potential loss of privacy, with this approach, we show that both the system and the user receive performance and security benefits.

### 3.4. Architecture for Spatiotemporal Blurring

We aim to blur time and location until there are at least *k* − 1 other users expected to be within the blurred time slot and region. There are at least three design alternatives to achieve spatio-temporal blurring. The first approach is to use a report-anonymization server, which collects each report (*t*, *l*, *d*), blurs it into (*s*, *r*, *d*), where *s* is a time slot and *r* is a region, and then forwards to the application. Although the architecture is simple, users must rely on the trustworthiness of the report-anonymization server for their privacy; the server must correctly blur every report to achieve the desired anonymity for all of the users and should be responsible for forwarding reports to appropriate applications without disruption. In the second approach, the blurring server receives a time-location pair (*t*, *l*) from an MN and responds with a blurred pair (*s*, *r*). The MN then submits a blurred report (*s*, *r*, *d*) to the application. Although the blurring server is unable to monitor or control report submissions, it can still manipulate the anonymization level of each report and can even target a specific user by selectively blurring reports. The third approach, which we chose for our architecture, introduces a map server (MS). The map server generates a map (one for each blurred time slot) that consists of blurred regions based on the population history and then distributes the maps to all of the MNs. The MNs can readily use the downloaded map for protecting their privacy without interacting with other entities. We only need to trust the map server for building maps correctly. In Section 6.1, we discuss the advantage of this map-server over a blurring server in more detail.

### 3.5. Assumptions

We make the following assumptions in our system. We assume that there is a mechanism for each MN to periodically submit a presence report, indicating its location to the map server, and each presence report can be anonymously authenticated, so that the server can build a population map [38]. See Section 6.3 for more detail. We assume that the MNs (and their carriers) trust the map server to properly construct the maps. We assume that the coverage of an access point is large enough to cover some public area in which anyone can possibly appear; otherwise, it may be trivial to link a presence in a certain location to a specific user. Our probabilistic *k*-anonymity scheme assumes that the population pattern during the training period (e.g., based on the past several days for some time of day) remains similar to the population pattern at the same time when the MN uses the map [39]. Our evaluation on real access point (AP)-association data (see Figure 2) shows that it is indeed a reasonable assumption that population patterns repeat everyday.

The APs are not trusted by the MNs to respect their privacy, but we assume that the map server can reliably authenticate anonymous users and their presence at a specific AP.

### 3.6. Design Goals

In the design of our spatio-temporal blurring scheme, our goal is to balance among three conflicting aspects of the scheme, namely privacy, utility and performance.

**Privacy:** As defined earlier, we seek to provide (*k*, *p*)-anonymity to users submitting reports.

**Utility:** We are concerned about the spatio-temporal utility of a participatory sensing system. In most applications (e.g., temperature sensing), it is desirable for the reported region to be compact (e.g., in a circular shape) and for the time range to be short. However, some applications require the region to be non-circular. For example, a traffic monitoring system may allow users to report their location by a road segment. In this case, the compactness is translated into the shortness of the segment. Our blurring scheme aims to be able to meet the utility requirements of a large range of applications (but not all) by blurring the region and the time only as much as is needed for the other design goals.

**Performance:** The computational overhead for producing the population-density maps (at the MS) or for using the population-density maps in blurring location and time in sensor reports (at the MN) should be reasonable with respect to the capabilities of the server and mobile devices, respectively.

We evaluate our approach on all three dimensions (privacy, utility and performance) in Section 5.

## 4. Spatiotemporal Blurring through Tessellation

Our scheme protects the location privacy of the users through two phases. In the first phase (the training phase), the map server (MS) builds a population map (for each time slot) so that each region of the map has at least *k* visitors during that time slot, with high probability. In the second phase, the MNs use the regions of the map when reporting their locations.

### 4.1. Population Map

A population map is a partitioning of the area map satisfying the following requirements. Given a positive integer *k* and a target probability *p*, the population map for a particular time slot *t* is said to be a (*k*, *p*)-population-map, or (*k*, *p*)-map in short, if every partition has at least *k* visitors during time slot *t* with probability no less than *p*. A time slot is an interval of time described by some start time and duration.

A (*k*, *p*)-map provides a probabilistic guarantee on the number of users in a certain area during a time slot. Our blurring scheme relies on the correct generation of such a map for each time slot. The MS may generate a map for each time slot by accumulating data over several days or weeks, then publishing the map for all MNs to download. Each MN blurs the time and location of its sensor reading based on the map; it submits a data report along with the time slot containing the time when the sensor data were collected and the partition containing the location where the sensor data were collected. With probability of at least *p*, the MN can hide itself among at least *k* − 1 other MNs.

### 4.2. Map Building

The MS builds a population map based on the area map, the AP locations, the MNs' anonymous presence reports and privacy parameters *k* and *p*. The output is a map tessellated into regions that aim to meet the (*k*, *p*)-criteria.

Below, we explain how the map is tessellated, how MNs report their locations and how the MS puts these together to get a (*k*, *p*)-map.

#### 4.2.1. Collecting Anonymized Presence Reports

To build a (*k*, *p*)-map, the MS needs a set of presence reports of the MNs appearing on the map. Specifically, each MN reports its location by a presence report (*s*, *l*) where *s* is a time slot and *l* is the identity of the currently associated AP. Every presence report is anonymously authenticated as assumed in Section 3.5 and described in Section 6.3.

The (*k*, *p*)-map captures the population pattern based on the collected presence reports. The duration of the collection depends on the temporal cycles of population density. In our experiments, the population pattern repeated on a daily basis, and we built the map based on ten days of presence reports.

#### 4.2.2. AP-Based Tessellation

The MS starts with a unit tessellation, composed of unit regions (called tiles), and merges some tiles to construct a coarser tessellation composed of sets of tiles (called clusters). The MS applies the (*k*, *p*)-criteria when it decides which tiles to merge. Therefore, the choice of unit tessellation is important for the map-building algorithm, because it affects how MNs can report their presences.

**Figure 1:**
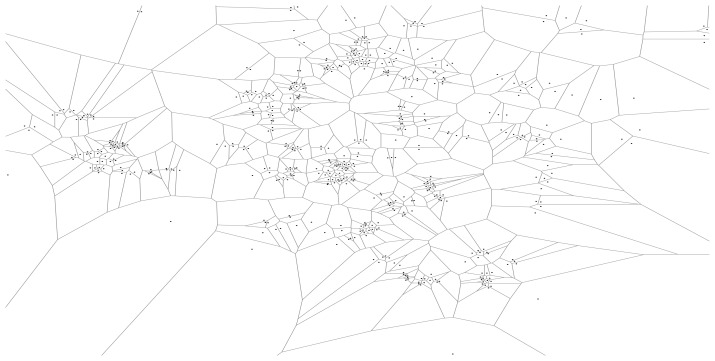
An example tessellation of all access points (APs)

We choose the Voronoi tessellation with respect to the Wi-Fi access point (AP) locations in the map as the unit tessellation. The AP-based Voronoi tessellation partitions the map into as many tiles as there are access points, and (except at the border) each tile is roughly as large as the coverage of that AP. The Voronoi tessellation divides the map into tiles, each containing one AP, such that any MN in a tile connects to the network through the AP associated with the tile. See Figure 1 for an example of AP-based tessellation. We denote an AP-based unit tessellation by 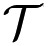 = {*t*_1_, *t*_2_, …, *t_n_*} where *n* is the number of APs, or tiles.

An alternative, often seen in the literature [40], is the use of regular grids as unit tiles. Using AP-centered Voronoi tiles as a unit tessellation has the following advantages. First, MNs can report their presences (by the currently associated AP's identity) without any special localization equipment, such as GPS. Second, AP-based population data are readily available in many WLAN systems for purposes such as load balancing, network management or security, which the MS can exploit for building population maps. Finally, the use of AP locations for tessellation meets our assumption that a unit region is large enough to cover some public area in which anyone can possibly appear (see Section 3.5).

#### 4.2.3. Map Building Algorithm

The map-building algorithm MAPGen in Algorithm 1 generates a (*k*, *p*)-map for each time slot *i* by iteratively merging tiles of the unit tessellation map 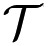 = {t_1_,… ,t_n_} into clusters to obtain a cluster set 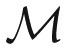
*_i_* = {*C*_1_, …, *C_m_*}, such that each cluster contains at least *k* visitors during the time slot *i* at least fraction *p* of the time.

At each round, we form a new cluster with the most-visited tile among unclustered tiles, then check whether the cluster meets our privacy criteria; that is, it has at least *k* visitors in at least *pN* time slots among *N* slots in total (Lines 5–11).



**Algorithm 1** MAPGen(*i*, *k*, *p*, 

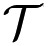
)
*Notation:* 

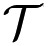
: the unit tessellation *S*: the set of all the *i*-th time slots V(*t*, *s*): set of visitors in tile *t* at time slot *s* iQuotient(*X*): isoperimetric quotient of region *X* Nb(*x*, *B*): *x* (tile or cluster) is adjacent to *B*
1:*B* ≔ ∅ ⊳Temporary cluster2:

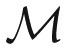
_i_ ≔ ∅ ⊳Output of the algorithm{Repeat until all tiles are processed}3:**while**

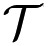
 ≠ ∅ or *B* ≠ ∅ **do**4:*  goodcluster* ≔ false {Pick the first tile for new cluster}5:  **if**

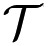
 = ∅ and *B* = ∅ **then**6:   
$t^{*}:=\underset{t\in T}{\arg\max}\underset{s\in S}{\sum |}\text{V}(t,s)|$7:   

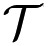
 ≔ 

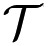
 \ {t*}8:   *B* ≔ *B* ∪ {t*}9:   **if** |{*s* ∈ *S*: Σ*_t_*_∈_*_B_* |V(*t*, *s*) | ≥ *k*} ≥ *p*|*S*| **then**10:   *goodcluster* ≔ **true**11:   **end if** {Pick a neighboring tile to merge}12:  **else if**

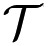
 ≠∅ ∧ *B* ≠ ∅ ∧ {*t*∈ 

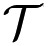
|Nb(*t*, *B*)} ≠ ∅ **then**13:   
$t^{*}:=\underset{\underset{\text{Nb}(t,B)}{t\in T}}{\arg\min}\text{iQuotient}(\{t\}\cup B)$14:   

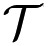
 ≔ 

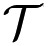
\{t*}15:   *B* ≔ *B* ∪ {t*}16:   **if** |{*s* ∈ *S* : Σ*_t_*_∈_*_B_* |V(*t*, *s*)| ≥ *k*} ≥ *p*|*S*| **then**17:   *goodcluster* ≔ true18:   **end if** {Merge with an existing cluster}19:  **else**20:   
$c^{*}:=\underset{\underset{\text{Nb}(C,B)}{C\in M_{i}}}{\arg\min}\text{iQuotient}(C\cup B)$21:   *B* ≔ *B* ∪ C*22:   

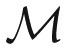
*_i_* ≔ 

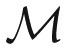
*_i_* \ {C*}23:   goodcluster ≔ true24:  **end if**25: v**if** goodcluster **then**26:   

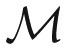
*_i_* ≔ 

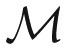
*_i_* ∪ {*B*}27:   *B*≔ **∅**28:  **end if**29:**end while**30:**return**

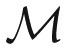
*_i_*


Otherwise, we merge this cluster with a neighbor tile so that the merged cluster is as compact as possible (think of a circle), so that it can represent a location better than non-compact ones (think of a long narrow rectangle) (Lines 12–18). We measure compactness using the isoperimetric quotient:

$$Q=\frac{4\pi A}{L^{2}}$$

where *A* is the area of the cluster and *L* is the circumference of the cluster. It is possible, however, that there is no neighboring tile left to add into the current cluster. In this case, we merge the current cluster with one of the neighboring clusters, such that the merged cluster is as compact as possible (Lines 19–22).

Our map-building algorithm runs in linear time with respect to the number of APs, *i.e.*, *O*(*n*).

### 4.3. Tessellation-Based Reporting

As described in the previous section, the map server generates a (*k*, *p*)-map for each time slot; let *N* denote the number of time slots per period. For example, if we choose an hour-long time slot with a daily period, then 24 maps will be generated. Each MN then anonymously downloads *N* up-to-date (*k*, *p*)-maps from the map server. Whenever the population pattern significantly changes, the map server needs to update the maps and re-distribute them to MNs accordingly.

When an MN wants to submit a data report containing its location and time, it identifies the map corresponding to the time and the cluster in the map corresponding to its current location. It then reports its blurred location-time by the map index, the cluster ID and the time-slot index of the current time. In this way, the MN can statistically achieve (with probability *p*) its anonymity among more than *k* − 1 other users, leaving a possible privacy risk (fewer than *k* − 1 other people around) with probability at most 1 − *p*.

## 5. Evaluation

It is infeasible to conduct a large-scale experiment with hundreds or thousands of users in a large-scale pervasive environment. Fortunately, we can model such an experiment using wireless mobility traces by interpreting associations with an access point as a user being in the area of that particular access point.

### 5.1. Dataset

**Figure 2:**
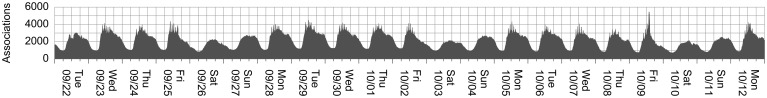
A histogram of association counts for every minute of our dataset.

**Figure 3:**
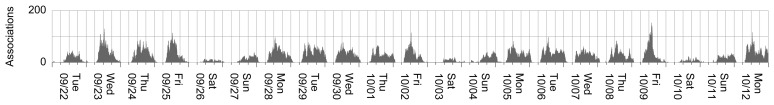
A histogram of association counts for every minute for the access point with the most associations

We conducted our experiments using association logs from the wireless infrastructure at Dartmouth College. The dataset represents the locations of a diverse population of students, faculty, staff, residents and visitors. The dataset contains 12,182 unique wireless clients making 730,943 associations with 717 access points between 22 September 2009 and 13 October 2009 (see Figures 2 and 3).

A wireless client averaged 60 total associations (min = 1; max = 1801; σ = 125) and visited an average of 11 unique access points (min = 1; max = 142; σ = 11). An access point averaged 1019 total associations (min = 1; max = 16,314; σ = 1288) and saw an average of 186 unique wireless clients (min = 1; max = 2381; σ = 254).

We flattened the access point locations to two dimensions by ignoring the “floor number” provided for each access point. As a result, some locations have tight clusters of APs as a result of multi-level buildings with APs at the same location on every floor; thus, we grouped APs that are within a short distance of each other into a single AP. We chose a distance of 5 m in our experiments, reducing the total number of APs to 486. Given this set of points on the plane, we generated a Voronoi diagram to produce a polygon for each AP (Figure 1).

**Figure 4:**
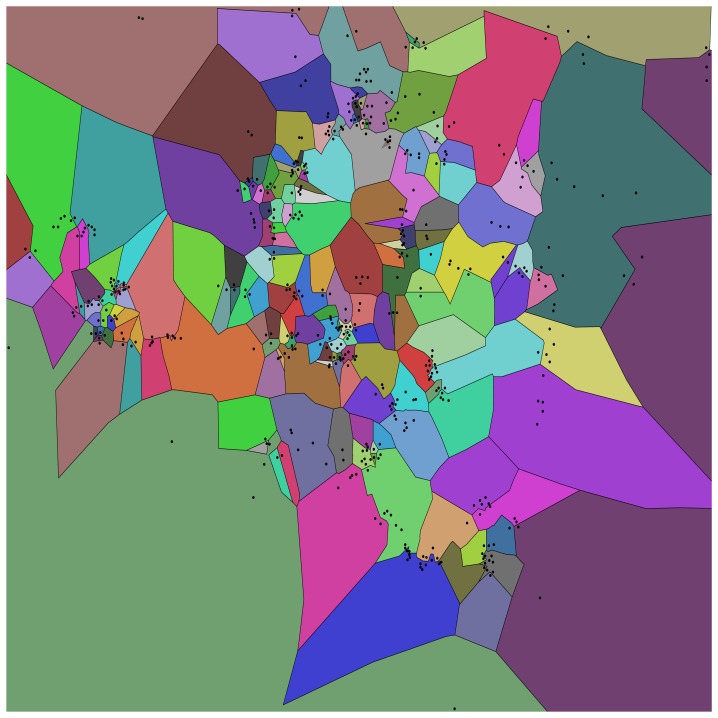
A (10, 0.7)-map generated for the 12 p.m.–1 p.m. time slot. Each colored region means that on 70% of the days, there were 10 or more unique associations between the hours of 12 p.m.–1 p.m. for each day between 22 September 2009 and 1 October 2009. The black dots correspond to AP locations.

Figure 4 shows an example of a (10, 0.7)-map generated from 10 days of history at the 12 p.m.–1 p.m. time slot. The points within the tiles indicate the locations of access points that were clustered together for that tile. There are 182 tiles in Figure 4, the smallest and largest being 89 m^2^ and 6,869,523 m^2^, with a median area of 2306 m^2^ (quartiles *Q*_1_ = 708 m^2^, *Q*_3_ = 7849 m^2^).Note that the tiles (and hence, clusters) near the edges of the map tend to have a large area, because we do not crop the tiles to the campus area.

There are many parameters that can be adjusted in the map-building algorithm. In general, tuning each parameter requires balancing the target *k*-anonymity with some utility. Table 1 shows the parameters used in the experiments. We assume utility is defined to be the median cluster size, as a smaller area plays a better role for the location in most applications. On the other hand, given a (*k*, *p*)-map and some future association data, we measure the achieved anonymity level of each map by *k*-accuracy: the ratio of the number of clusters that had at least *k* people to the total number of clusters. That is,

$$k-\text{accuracy}=\frac{\left|\{C\in M|u(C)\geq k\}\right|}{\left|M\right|}$$

where, given a time slot, 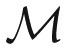 is the cluster-set generated by the map-building algorithm, and a function *u*(*C*) is the number of users who appeared in cluster *C* during the time slot. *k*-accuracy is a posterior metric that reflects how well a (*k*, *p*)-map achieves the privacy goal denoted by *k* and *p*. Intuitively, there is a trade-off between privacy represented by *k* and utility represented by the cluster size. We can balance anonymity and utility by tuning the parameters of the map.

On average, it takes 4 s to compute a map, with about 1 s spent on actual clustering (using a 2.93-GHz Intel Core i7 with 8 GB of 1333-MHz DDR3 memory). For each map, we computed the median cluster size and examined the next seven days after the history interval to compute the k-accuracy for each of these future dates.

### 5.2. Target k vs. Probability p

**Figure 5:**
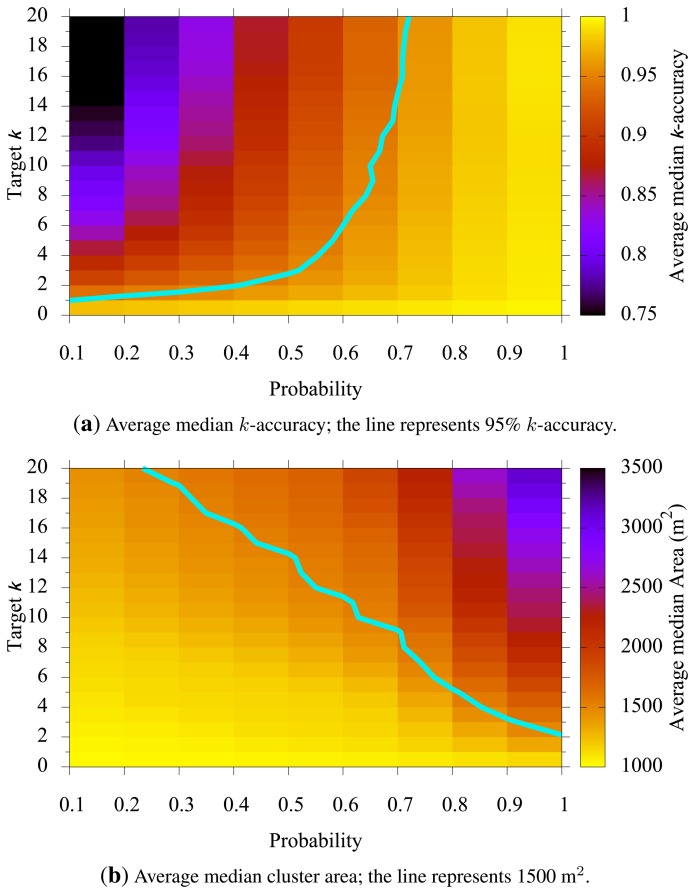
Target *k vs.* probability *p*.

Figure 5 shows how a chosen target *k* and probability *p* affected *k*-accuracy and the median cluster size. In general, if target *k* increases, we enlarge each cluster to observe more users therein, and thus, we have higher *k*-accuracy (as *u*(*C*) becomes higher). The same is expected as the probability *p* increases. Figure 5a clearly shows the positive correlation between *k* (or *p*) and *k*-accuracy. The line in the figure represents where *k*-accuracy is 95%. Note that a probability of 70% or higher yields *k*-accuracies of 95% or better regardless of the chosen target *k*. This is so because we may end up with a larger cluster size than the ideal cluster size to meet the probability criteria. Figure 5b also shows the positive correlation between *k* (or *p*) and the median cluster size. These two graphs combined, we can determine parameters *k* and *p* based on the privacy goal (*k*-accuracy) and the utility goal (cluster size).

### 5.3. Target k vs. Time

**Figure 6:**
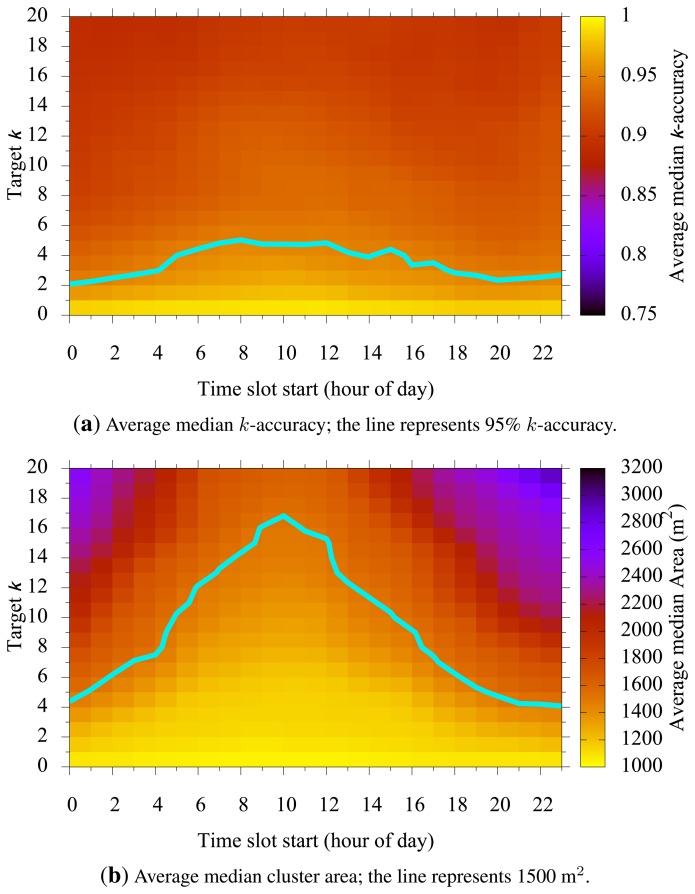
Target *k vs.* time slot start.

Figure 6 shows how a chosen target *k* and time slot start affecting *k*-accuracy and cluster size. In Figure 6a, we see that the *k*-accuracy is relatively stable regardless of the chosen time slot start. This is desirable, as it implies that the same level of privacy is maintained throughout different time slots in a day. However, the cluster size showed notable differences among different slot times. Depending on the selected time slot start, Figure 6b shows us that for a given time slot and target *k*, the cluster size increased for those times when the population is relatively inactive. The cluster size, on the contrary, decreases as the time slot moves toward high population periods. During high population times, around noon for example, smaller clusters can meet the privacy requirement, resulting in better utility.

### 5.4. Time Slot Start vs. Time Slot Duration

**Figure 7:**
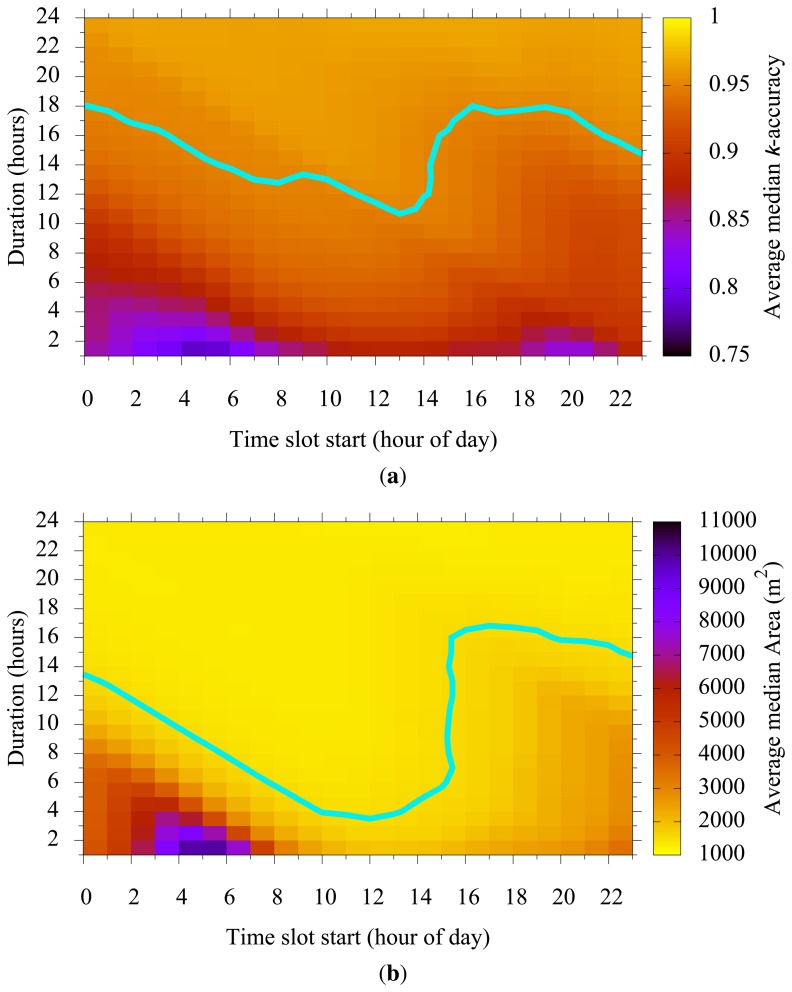
Time slot duration *vs.* time slot start. (**a**) Average median *k*-accuracy; the line represents 95% *k*-accuracy; (**b**) average median cluster area; the line represents 1500 m^2^.

Figure 7 shows how a chosen time slot start and duration affected *k*-accuracy and cluster size. Figures 7 show that as the time slot start moves from low population time (e.g., midnight) toward the high population time (e.g., 2 p.m.), the slot duration necessary to get high k-accuracy and a small cluster size decreases. We also observe an irregular increase of necessary slot duration due to the time slots spanning across consecutive days.

The time slot duration reflects the delay tolerance of an application. If an application requires the user to submit a report within an hour of generation, we can use the slot duration of an hour. The k-anonymity set then contains only those who appear within that duration. However, if the application allows a 10-hour delay for report submission, the user has a 10-h long period to mix itself with other users appearing in the same period. We prepare population maps with different time-slot durations so that the user can choose the map that best fits the application. We can also utilize these figures to tune parameters (e.g., *p* or *k*) for a particular delay-tolerance requirement of an application.

### 5.5. Choosing Parameters for Privacy and Sensing Quality

Our blurring mechanism uses two parameters, *k* and *p*. These parameters determine the level of user privacy; for larger *k*, users enjoy higher anonymity, and for larger *p*, the claimed anonymity is more likely to be achieved. Higher *k* or *p*, however, may cause a larger cluster size, resulting in imprecise location reports. For example, an MN may report a temperature value, but along with a large area for the sensing location. Therefore, and as shown in Figures 5, 6 and 7, there is a trade-off between privacy and quality of sensing in choosing *k* and *p*.

In this regard, it is important to know the largest acceptable cluster size for the specific sensing application. For example, studies show that a coverage of 3533 m^2^ suffices for sensing temperatures in urban area [28], while some dense city areas with complex structures (e.g., downtown Tokyo) may require no more than 537 m^2^ per sensor [5]. On the other hand, it has been reported that in cellphone-based traffic monitoring systems, it suffices to have location accuracy of about 100 m for correct localization of vehicles [41]. This implies that a cluster size of up to 31,400 m^2^ could be acceptable for reasonably accurate traffic estimation.

Another factor on sensing quality is the temporal accuracy of the reports. The temporal blurring occurs by given time slot duration. For a one-hour duration, every report generated within that time frame is annotated by the time slot range as its sampling time. This temporal accuracy is relatively easy to deal compared to locational accuracy; just pick the map generated with an appropriate time slot duration. Note that the map server prepares maps with various time slot durations for this purpose.

In addition to quality of sensing, choosing the right *k* value is related to the perception of privacy by the user. This perception is to some extent personal or situation dependent. For example, some users may feel comfortable with *k* ≥ 10, while others are happy with *k* ≥ 5. Or the same user may prefer larger *k* for certain situations. The proposed system, however, uses a global *k* value, as a lower bound for all participating users. Studies show that common practice in medical data release is to use *k* ≥ 5, and it is quite rare to use *k* ≥ 15 [42].

## 6. Discussion

In this section, we discuss some issues regarding the proposed scheme.

### 6.1. Online vs. Offline Map Server

Instead of building population estimate maps for offline use, users could obtain real-time presence information from an online MS. We list several drawbacks of such an online approach relating to performance and security.

Latency and energy consumption: For each report, MNs must query an online MS for every location and time period when reports were collected; this introduces more latency between when reports are collected and when they are delivered. Furthermore, each such query must be made through a new connection through a mixed network (to prevent the MS from linking the individual reports together), adding additional delay each time a report is submitted (and each such query must be delayed to the end of the time range). With an offline MS, MNs make a local hash-table lookup, which is nearly instantaneous. Because of these reasons, the energy consumption at the MN is much lower for an offline server.

Bandwidth consumption: In the offline MS case, maps are relatively small and are downloaded once per time period that an MN is active. In contrast, an online approach requires a secure connection setup each time, which could result in more bandwidth usage depending on how many times the MN creates a report. In the offline case, the mapping of APs to partitions needs to be communicated once, whereas in the online case, every query could result in a new, different partition that is defined by a list of APs.

Scalability and complexity of server operation: An offline server can manage scalability better, since a central offline MS can offload distribution of maps to replicated servers. An online MS, on the other hand, must ensure the consistency of information if replicated servers are used. Furthermore, an online MS must continuously update the map as location reports come in or recompute the map from scratch every time a query arrives. Such operation would place a much higher burden on the online MS and would be more complex to implement.

Trust tradeoffs: With an online MS, the MN's owner must trust the MS not to track the MN's location by observing the series of locations mentioned in its queries. With an offline MS, we assume that the MS receives anonymized visitation data from the network APs or network operator; the carriers need not trust the MS with specific location history data.

Privacy tradeoffs: An online MS can attack any subset of querying MNs by producing incorrect counts to a querying MN and, thus, violate the privacy of MNs through clusters that do not provide the requisite anonymity. The MS could provide different counts or clusters to different queriers (with the same time-location query), thus making the reports recognizable when the reports are filed, allowing the MS to link the query to the report. With an offline map-building approach, however, the MS must publicize the map and try to attack everybody, thus increasing the likelihood of getting implicated in such an attack. Furthermore, MNs can consult the map locally and decide when to or when not to report without needing to query the MS. Accuracy tradeoffs: For all its disadvantages, an online MS provides real-time and accurate knowledge of anonymity counts for clusters, whereas an offline MS provides only a statistical guarantee. Given all of the advantages of an offline MS listed above, our goal then was to evaluate the performance of an offline MS in providing such statistical guarantees. This evaluation forms the main contribution of our work.

### 6.2. Personalized k-Anonymity

One natural suggestion would be to allow users to specify their desired degree of *k*-anonymity and pick the right cluster to match the specified *k*. This approach can result in a privacy leak, however, because the user's preferred degree of anonymity can itself reveal information about the user. Consider the example where a known paranoid user prefers *k*-anonymity with *k* = 200. Receiving a report with a large blurred range can be linked to that user. Users' privacy is therefore maximized when all users use the same value of *k*. Nevertheless, the system could offer multiple maps for a small set of *k* values so that users can locally choose their map of the preferred *k* value.

### 6.3. Anonymous Authentication

To build the population-density maps, which describe the “typical” density of mobile devices in each area of the network at a given time of day, the map server (MS) needs to collect historical location information from all MNs in the system. We assume, therefore, that the MS has access to a feed of MN location observations produced by the wireless-network infrastructure. Many enterprise Wi-Fi networks, for example, use a RADIUSserver to authenticate every device attached to the network. If, as in most such enterprises, the RADIUS server is trusted with the identity of the devices, it could be additionally trusted with the task of de-identifying this information before feeding it to the map server.

If stronger privacy guarantees are desired, we imagine a modified RADIUS server that uses anonymous authentication protocols. The details are beyond the scope of this paper, but consider the following approach. First, we divide time into intervals of a certain granularity (e.g., five minutes). When the MN connects to the network, it proves to the RADIUS server that it is authorized to use the network without disclosing its identity [38]. We arrange that an MN's authentications across different time periods are unlinkable, but if the MN authenticates at multiple access points within the same time interval, the system can recognize the MN as the same MN at those access points. In other words, authentications are pseudonymous within time intervals, and anonymous across time intervals. Such authentication can be realized through n-times periodic anonymous authentication [38], where the parameter *n* specifies how many anonymous authentications are allowed (in this case, *n* = 1) per period (time interval).

### 6.4. Location Data and Tessellation

We describe our tessellation approach using data about the location history of wireless-network users, which is provided as a sequence of associations with Wi-Fi APs. The movements are discrete, hopping from one location to another, and the locations are discrete points on the plane. In other settings, such as locations obtained from GPS, the locations are continuous (any coordinate on the Earth), and the movements may be less discrete (a path connecting waypoints). We believe that our tessellation approach can be adapted to other location models, such as heat maps showing continuous mobility distribution, although the details remain future work.

## 7. Related Work

Borrowing from the data privacy literature, most researchers on location privacy of mobile users employ the principle of *k*-anonymity [16] as their privacy model, called location *k*-anonymity [17–24].

In the context of location-based services (LBS), Gruteser and Grunwald [17] first adopted *k*-anonymity for location privacy, proposing a quad-tree-based cloaking algorithm. The algorithm recursively divides the area down to the smallest rectangle that contains at least k users. Casper [21] used the pyramid structure to cloak user locations, and Kalnis *et al.* [23] proposed nearest neighbor cloak and Hilbert cloak algorithms for providing location *k*-anonymity. On the other hand, CacheCloak [22] mixes the user's predicted path with other users' paths, to confuse the adversary, and Hoh and Gruteser [24] perturb the user's path to confuse the paths of different users. However, it is possible that a large cloaked region significantly degrades the quality of the location-based service.

To address the quality degradation due to location blurring, Gedik and Liu [18,19] proposed a region-cloaking algorithm that supports both location privacy and QoS requirements on a per-user basis. Xiao *et al.* [20] also support both privacy requirements *k* and QoS constraints: max cloaking latency and max cloaking region size. Using a directed graph, their scheme achieves both location anonymity and identifier anonymity. To promote the success rate of cloaking, the scheme introduces dummy locations. Our proposed scheme can fine-tune the privacy and quality trade-off using the parameter *p*: with smaller *p*, we have better QoS, but degraded privacy.

Most location privacy schemes (as introduced above) adopt a centralized approach where a dedicated and trusted anonymization server mediates between the mobile device and the service (*i.e.*, LBS server). The anonymization server analyzes requests (queries, reports) and modifies them if necessary before forwarding to the service. However, a centralized approach has limitations in both performance and security (as we note in Section 1). To alleviate this problem, our scheme introduces the map server, instead of an anonymization server, which provides statistical guidelines to the user so that the users can anonymize their reports by themselves. Our solution requires a smaller trusted computing base, and the users retain more control over privacy.

Alternatively, some decentralized approaches have been proposed. These approaches eliminate the requirements of an intermediary trusted server by letting user nodes communicate with each other directly, collectively playing the anonymization server's role [25,26,43–46]. For example, Solanas and Martinez-Balleste [44] proposed *k*-anonymity based on peer-to-peer (p2p) communication, in which local peers find each other, form an anonymity set of larger than *k* and compute the center of their locations. To hide their locations from peers, they use public-key privacy homomorphism. Hashem and Kulika [43] proposed a linear-time algorithm for generating a user's *k*-anonymous imprecise location without revealing exact locations. Prive [26,45] uses Hilbert curves to partition users into local groups and then forms an overlay network to cloak user location without a central server, while Hu and Xu [46] proposed to use only proximity information between users for location cloaking, without revealing the exact locations of the users. Recently, cloud-based crowd-sensing mechanisms were proposed [47,48]. In [48], the cloud agents collaborate with other agents in a p2p fashion, to achieve the desired privacy while providing data to the data consumers.

Unfortunately, the p2p approach requires *k* − 1 other users to be online (*i.e.*, present and able to communicate) in the user's vicinity. Ideally, users should achieve *k*-anonymity even if the other *k* − 1 users visit the same area at a later point within a particular time window (in this regard, a server-based approach can combine reports from users who may have visited a particular location at different times). In this regard, our scheme does not require the users to be simultaneously present in the same region in order to form an anonymity set. Instead, it suffices for them to appear in the same time slot.

Another problem with the p2p approach is that the *k* − 1 users may be located beyond the range of direct communication (e.g., if Bluetooth is used). Mokbel and Chow outline several challenges [49] in this space and suggest a multi-hop approach to reach such nodes [25]. Their approach, however, assumes that nodes trust other nodes with their location information. Ideally, the p2p protocol should require the nodes to anonymously exchange and combine reports while preserving privacy. Secure multi-party computation [50] provides such solutions, but it is computationally expensive and requires the *k* parties to be online simultaneously, neither of which may be acceptable for mobile, pervasive devices. In our approach, nodes can individually and efficiently compute their own *k*-anonymous location by table lookup in a compact, occasionally downloaded, anonymization map, never sharing their actual location with anyone or any server.

Turner *et al.* [51] proposed a dynamic tessellation algorithm, called Anonoly, which provides a mechanism for the *k*-anonymity tessellation map to adapt to the change in report population. Although their approach resembles ours, Anonoly's privacy goal is different from ours. While we support *k*-anonymity with respect to the number of users at a tile, Anonoly cares about *k*-anonymity with respect to the number of reports. It is easy to count the number of reports in a tile on the fly. On the other hand, it is difficult to count the number of users in a tile, as not all of the users will submit a report. However, our scheme adapts to the change of population over time by providing different maps for each time slot, instead of adapting to the instantaneous changes in population. By periodic updates to the map, our population map follows the long-term trend of the population change.

## 8. Conclusions

We present a novel method for mobile crowd sensing systems to provide *k*-anonymity with respect to user locations. Using our technique, users' devices can automatically blur time and location information in reports without needing a fully-trusted centralized server with statistical *k*-anonymity. We evaluated our approach using real AP-association traces and show that a reasonable tradeoff can be achieved between users' privacy and spatiotemporal granularity.

## Figures and Tables

**Table 1:** Experiment parameters.

**Parameter**	**Values**
Map-building days	10 days
Target *k*	0–20
Probability *p*	0.1–1.0
Time slot duration	1–24 h
Time slot start	12 a.m.–11 p.m.
